# A new type of solar-system material recovered from Ordovician marine limestone

**DOI:** 10.1038/ncomms11851

**Published:** 2016-06-14

**Authors:** B. Schmitz, Q. -Z. Yin, M. E. Sanborn, M. Tassinari, C. E. Caplan, G. R. Huss

**Affiliations:** 1 Astrogeobiology Laboratory, Department of Physics, Lund University, 221 00 Lund, Sweden; 2 Hawai'i Institute of Geophysics and Planetology, University of Hawai'i at Manoa, Honolulu, Hawaii 96822, USA; 3 Department of Earth and Planetary Sciences, University of California at Davis, Davis, California 95616, USA

## Abstract

From mid-Ordovician ∼470 Myr-old limestone >100 fossil L-chondritic meteorites have been recovered, representing the markedly enhanced flux of meteorites to Earth following the breakup of the L-chondrite parent body. Recently one anomalous meteorite, Österplana 065 (Öst 65), was found in the same beds that yield L chondrites. The cosmic-ray exposure age of Öst 65 shows that it may be a fragment of the impactor that broke up the L-chondrite parent body. Here we show that in a chromium versus oxygen-isotope plot Öst 65 falls outside all fields encompassing the known meteorite types. This may be the first documented example of an ‘extinct' meteorite, that is, a meteorite type that does not fall on Earth today because its parent body has been consumed by collisions. The meteorites found on Earth today apparently do not give a full representation of the kind of bodies in the asteroid belt ∼500 Myr ago.

## Abstract (web summary)

Meteorites falling on Earth today are believed to represent 100–150 parent bodies. Within 470  Myr ago sediments at a limestone quarry in Sweden, Schmitz *et al*. have found and identified a new type of meteorite based on chromium and oxygen isotopes sourced from a previously unknown parental body.

## Graphical abstract

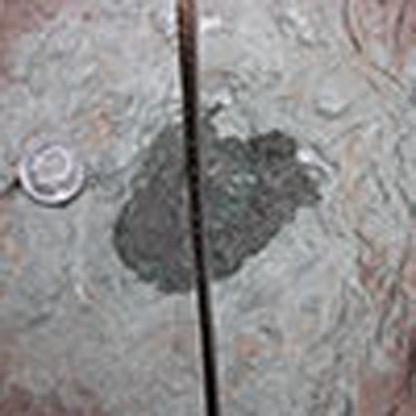

The ordinary chondrites are the dominant type of meteorite falling on Earth today, representing about 85% of the total flux. They are divided in three subgroups, H (42% of ordinary chondrite falls), L (47%) and LL (11%), depending on their iron and metal content. In the 1960s, it was realized that the L chondrites show a major peak in K–Ar degassing ages at **∼**500 Myr ago^1^,^2^, an age recently better constrained to **∼**470 Myr ago^3^. These meteorites apparently underwent a massive collisional event at this time, involving the breakup of their parent body. In contrast, the typical degassing age of most meteorite types goes back to the young solar system, that is, >3.5 Gyr ago. In marine limestone that formed on Earth around 470 Myr ago, there is an evidence for an at least two orders of magnitude increase in the flux of L-chondritic meteorites and micrometeorites (for a review, see ref. 4). The prime evidence is the more than 100 fossil L chondrites recovered during the industrial quarrying of Ordovician limestone in southern Sweden^4^,^5^. The meteorites are almost completely replaced by secondary minerals, but spinel grains, and often also petrographic textures, are preserved. Mineralogical, chemical, isotopic and petrographic studies have shown that mid-Ordovician fossil extraterrestrial material is indeed of L-chondritic origin^4^. Recently, one 8-cm-large meteorite was found, containing spinel grains very different from all the other meteorites^6^. On the basis of ^21^Ne cosmic-ray exposure ages of its spinel grains this meteorite, Österplana 065, previously referred to as the ‘mysterious object', appears to have been liberated as a meteoroid at the same time as the other fossil meteorites^6^,^7^,^8^. It may thus represent a piece of the impactor that broke up the L-chondrite parent body^6^. The breakup of this body and the following enhanced flux of extraterrestrial material, including km-sized asteroids, coincide with marked evolutionary changes in Earth's invertebrate life, the so-called Great Ordovician Biodiversification Event^9^. Unravelling why the L-chondrite parent body broke up at this time, may lead to a better understanding of possible large-scale astronomical perturbations affecting both Earth and the solar system^4^.

Österplana 065 was preliminarily classified as ‘winonaite-like', based on oxygen isotopic and elemental composition of its spinels and by comparison with a winonaite clast in the Villabeto de la Peña ordinary (L6) chondrite^6^,^10^. In order to further clarify the origin of Öst 65 here we apply chromium isotopic analyses of whole-rock samples of Öst 65 plus three fossil L chondrites (Österplana 018, 029 and 032, or informal names Ark 018, Gol 001 and Bot 003, respectively), as well as clast and host material from Villabeto de la Peña. We also perform Cr-isotopic analyses of separated chrome-spinel grains from two recent meteorites, the winonaite NWA 725 and the L6 ordinary chondrite Lundsgard. In addition, we present refined oxygen three-isotopic analyses for the chrome spinels of Öst 65, and new data on the appearances and distribution of relict minerals in Öst 65. We show that Öst 65 has no documented analogue among the known meteorites that have fallen on Earth in recent time. The ^54^Cr values of Öst 65 are similar to those of ordinary chondrites, whereas oxygen isotopes show its affinity with some rare primitive achondrites.

## Results

### Isotopic results

**Figure 1:**
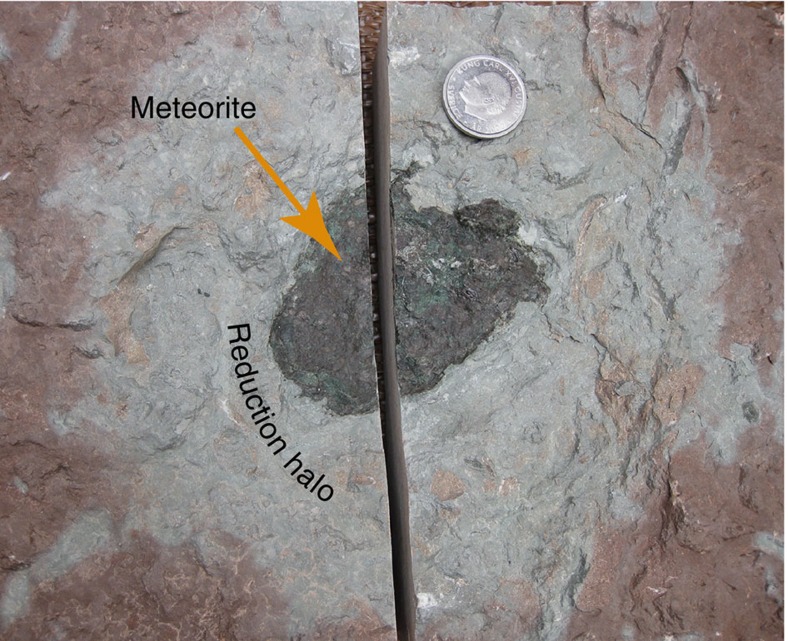
The Österplana 065 fossil meteorite from the Glaskarten 3 bed. The meteorite is 8 × 6.5 × 2 cm large. It is surrounded by a grey reduction halo, in the otherwise red limestone. Oxygen was consumed when the meteorite weathered on the sea floor. The coin in the image has a diameter of 2.5 cm.

**Figure 2:**
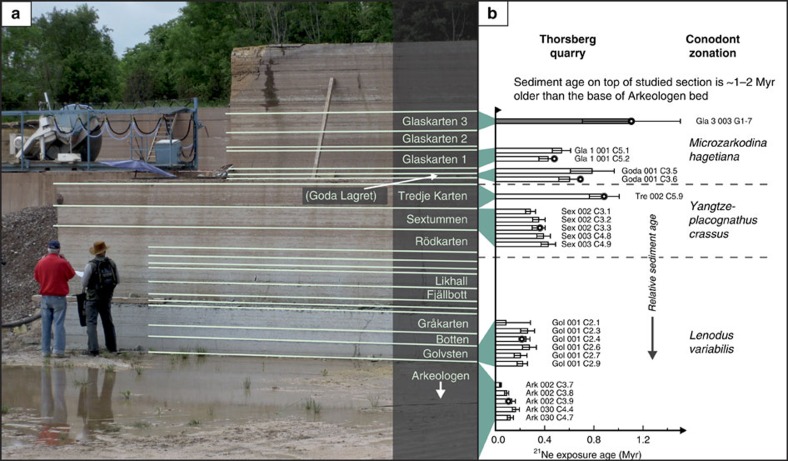
Thorsberg quarry and cosmic-ray exposure ages of fossil meteorites. (**a**) The quarried profile in the Thorsberg quarry with the names of individual beds. (**b**) Cosmic-ray exposure ages for some of the mid-Ordovician fossil meteorites. Meteorite cosmic-ray exposure ages increase with stratigraphic height, in accordance with sedimentation rates as established from conventional biochronology^7^,^8^. The data indicate that all the fossil meteorites, including Öst 65, originate from a breakup event that took place about 200 Ka before the lowermost beds in the quarry formed^6^. The details and the analytical background of the ^21^Ne exposure ages are discussed in ref. 6. In the figure the Österplana 065 meteorite is represented by its ‘field' name, Gla3 003, at the top of the section studied. Our fossil meteorites obtain official names, approved by the Meteoritical Society, following the convention of naming meteorites after the locality at which they have been found, that is, in this case Österplana. But in order to emphasize that meteorites in different beds fell at different times they also receive an informal ‘field' name. For Öst 65 the name Gla3 003, implies that it is the third meteorite found in the bed Glaskarten 3.

**Figure 3:**
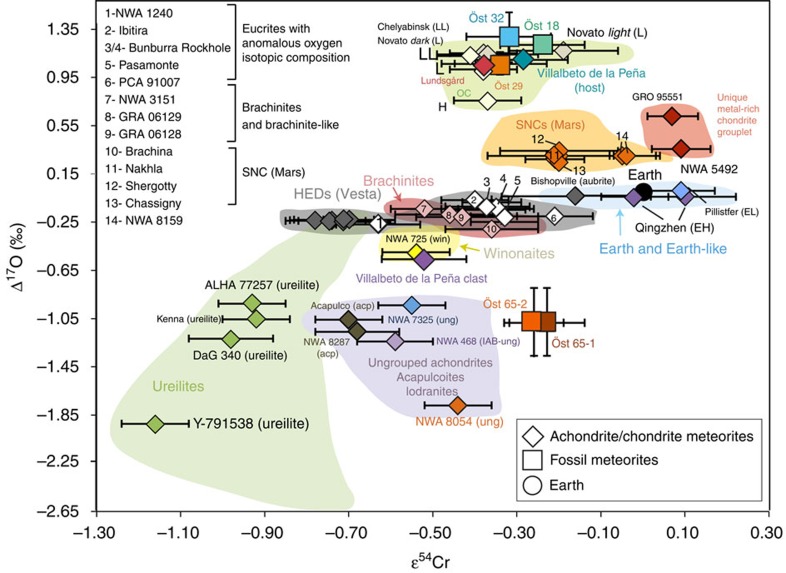
Oxygen and chromium isotopic composition of meteorites. Comparison of Δ^17^O versus ɛ^54^Cr of achondrites, and ordinary chondrites fallen on Earth in recent time with Österplana 065 (Öst 65) and fossil L-chondritic meteorites Österplana 018, 029 and 032 (Öst 18, Öst 29 and Öst 32 in figure). The fields for ordinary chondrites (OC), Mars (SNCs), Earth and earth-likes, Vesta (HEDs), brachinites, ureilites, winonaites (win), acapulcoites (acp)/lodranites/ungrouped achondrites (ung) and a newly identified unique metal-rich chondrite are marked with select representative samples with available data. Symbol colours indicate meteorite type or grouping. Note that the carbonaceous chondrites and affiliated achondrites plot outside the field, with highly positive ɛ^54^Cr. The ɛ^54^Cr literature values are from refs 12, 18, 19, 24, 25, 26, 27, 28, 29, 30, 31, 32, 33, and Δ^17^O literature values are from refs 10, 12, 25, 30, 34, 35, 36, 37, 38, 39, 40, 41, 42, 43.

The fossil meteorite Öst 65 is shown in Fig. 1, and its stratigraphic position in the Thorsberg quarry, together with previously established ^21^Ne cosmic-ray exposure ages^6^,^7^, is presented in Fig. 2. Our new ɛ^54^Cr and Δ^17^O data are compared to similar data for many of the major groups of meteorites known today in Fig. 3. For the detailed results of our isotopic analyses, see Tables 1 and 2. The spinel grains from Lundsgard and NWA 725 yield ɛ^54^Cr values that are the same as previously analyzed L6 chondrites and winonaites, respectively. The samples of the fossil L chondrites yield ɛ^54^Cr results in agreement with an ordinary chondrite origin, that is, in the range −0.34 to −0.24. This indicates that whole-rock analyses of fossil meteorites provide reliable Cr-isotopic results. This is in line with previous elemental analyses of whole-rock fossil L chondrites showing that they have retained most of their original chromium^11^. The Villabeto de la Peña host and clast give ɛ^54^Cr values confirming the previous classifications based on oxygen isotopes alone^10^. The two whole-rock samples of Öst 65 yield ɛ^54^Cr values of −0.26 to −0.23, which are within the uncertainties of previously measured ordinary chondrites^12^. The new oxygen-isotope analyses confirm previous Δ^17^O values for Öst 65 spinels just below the terrestrial fractionation line. The data plot with the winonaites, acapulcoites and lodronites, clearly separated from the chromites of the typical L-chondrite fossil meteorites. Although the oxygen isotopes support a relationship to the winonaites (as suggested in ref. 6), acapulcoites or lodronites, the ɛ^54^Cr results for Öst 65 rule out such a relationship. Our combined ɛ^54^Cr and Δ^17^O analyses of Öst 65 suggest that it originated from an asteroid of a kind not represented in our collections of recent meteorites (Fig. 3).

### Mineralogy and petrology

**Figure 4:**
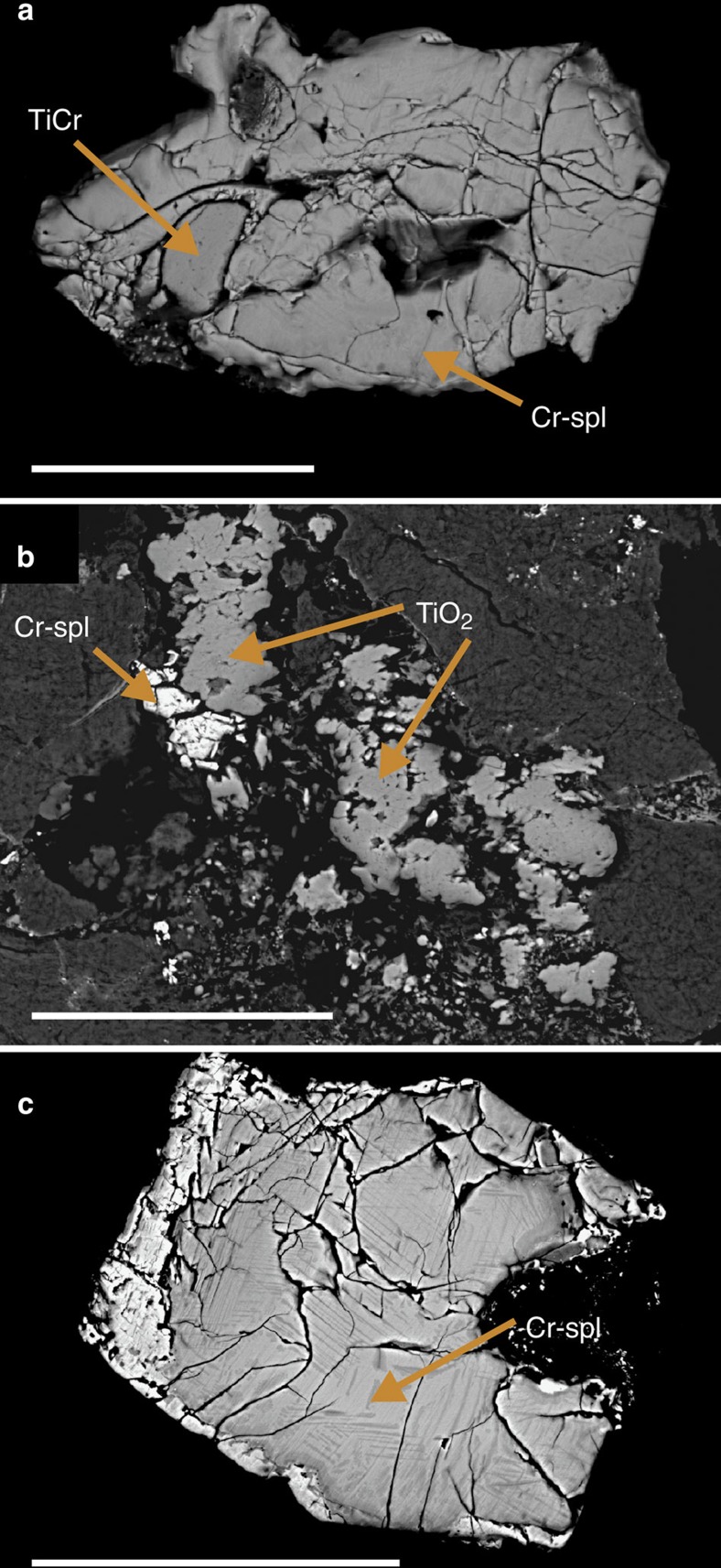
Back-scattered electron images of mineral grains from Österplana 065. (**a**) Chrome-spinel grain with inclusion of a TiCr-mineral, possibly olkhonskite (TiO_2_=56 wt%, Cr_2_O_3_=30 wt%, FeO=9 wt%). (**b**) Rutile and chrome spinel in polished section of Öst 65. (**c**) Chrome-spinel grain with apparent shock deformation lamellae. Scale bars, 50 μm.

Mineralogical and petrological data support the conclusion that Öst 65 represents a so far unknown type of meteorite. Our data rule out that Öst 65 is a breccia with clasts of, for example, winonaite and ordinary chondrite origin. The relict texture is unbrecciated and no relict chondrule texture is observed. We have dissolved several fragments from different parts of Öst 65, and have not been able to recover one single grain of the type of chromite that is so common in equilibrated ordinary chondrites. Such chromite (>ca. 30 μm) has a narrow element compositional range, making it easy to identify^4^. Our studies of polished sections show that the type of chrome-spinel grains we used for oxygen isotopic analyses are distributed throughout the meteorite. There are no MgAl spinels (>5 μm) in Öst 65. The only type of inclusion found in the chrome spinels from Öst 65 has a Ti- and Cr-rich composition (similar to olkhonskite). These inclusions are quite common in the spinels of Öst 65 (Fig. 4a), but no silicate inclusions have been found although searched extensively. The chrome-spinel grains are sometimes associated with 10–30 μm large rutile grains (Fig. 4b). To our knowledge there is no known meteorite with the chrome-spinel–rutile assemblage that we observe in Öst 65.

The majority of the chrome-spinel grains in Öst 65 show abundant planar deformation features (Fig. 4c), something not observed in the many fossil L chondrites. The planar features appear similar to shock lamellae in chromites from shock melt veins in recent meteorites^13^. The Öst 65 spinels are also heavily fractured (Fig. 4a,c), which provides independent evidence for severe shock. Chromites of recently fallen L chondrites show increasing levels of fracturing with increasing shock stage^14^.

## Discussion

The meteorite Öst 65 accompanies >100 L chondrites recovered in the sediments of the Swedish quarry. Although single random meteorites are possible, one has to consider that Öst 65 represents on the order of one per cent of the meteorites that have been found on the mid-Ordovician sea floor. This indicates that Öst 65 may represent one of the dominant types of meteorites arriving on Earth 470 Myr ago. Although we cannot rule out that a meteorite similar to Öst 65 may eventually be found among meteorites falling on Earth today, we can be quite certain that the Öst 65 type does not represent one per cent of the 52,600 classified recent meteorites. At the most there could be a few overlooked specimens.

The discovery in the Ordovician record of a meteorite type that is apparently not falling on the Earth today has important implications. For example, it is possible that the asteroid that produced Öst 65 no longer exists, and there is no source for such meteorites today. The asteroid belt has been evolving through collisions over the history of the solar system, and many of the original asteroids have undoubtedly been destroyed. The record of fossil meteorites on Earth (or on other planetary bodies) provides the only evidence for their former existence and the only way to investigate the collisional evolution of the solar system. The diversity of meteorite parent bodies even relatively late in solar-system history, ∼500 Myr ago, may have been significantly greater than today. We know, for example, that remnants of Earth building blocks are not present in our meteorite collections. Such meteorites are extinct now but clearly built the Earth in the distant past.

The cosmic-ray exposure age of Öst 65 is ∼1 Myr and is within the range of those of the fossil L chondrites (Fig. 2)^6^,^7^,^8^. This associates Öst 65 with the breakup of the L-chondrite parent body. The high level of shock metamorphism in the chrome-spinel grains (Fig. 4c) is consistent with the Öst 65 parent body having experienced a major collision. It is possible that a collision between the L-chondrite parent body and the Öst 65 parent body resulted in almost complete destruction of the Öst 65 parent asteroid while only disrupting the L-chondrite parent body into several smaller bodies and debris. These smaller bodies continue to feed meteorites, but there is nothing left to provide Öst 65 meteorites to Earth today.

We do not know whether the Öst 65 parent body was one of the original asteroids or already a fragment of a larger object. Iron meteorites come from parent bodies that experienced melting and differentiation that separated the Fe–Ni metal from the silicate components. Trace-element data indicate that iron meteorites may originate from up to 60 different parent bodies^15^. With a few potential exceptions, we have not been able to identify the silicate fractions that accompanied the Fe–Ni metal in the original parent bodies. The silicates were probably stripped from tougher iron cores of the asteroids early in solar-system history. The iron cores of these parent bodies were much more resistant to collisional degradation, whereas the outer silicate parts have been consumed over time in this process^16^. Because Öst 65 does not exhibit chondrite texture and has other characteristics that do not match most chondrites, the Öst 65 parent body might represent some of this missing silicate. Winonaite meteorites have very similar oxygen-isotope compositions to the silicate inclusions in IAB and IIICD iron meteorites, implying that they may have originated on the same parent asteroid^15^,^17^. The ‘forensic' power of paired Δ^17^O-ɛ^54^Cr isotope systematics^18^,^19^ could be applied to silicate and oxide inclusions of many types of iron meteorites in order to identify their genetic link with other planetary materials. Studies of fossil meteorites in the Earth sedimentary record may identify other classes of ‘extinct' meteorites, some of which could be linked to known classes of iron meteorites.

The Öst 65 meteorite is significant because it demonstrates that ∼500 Myr ago we may have had different meteorites falling to Earth than what we see today. Some may be extinct, whereas others, such as the L chondrites, still fall on Earth. Apparently there is potential to reconstruct important aspects of solar-system history by looking down in Earth's sediments, in addition to looking up at the skies^4^.

## Methods

### Chromium isotopic analysis

Aliquots for Cr-isotopic composition measurements were prepared using bulk material for seven of the samples: Öst 65-1, Öst 65-2, Öst 18, Öst 29 and Öst 32, a winonaite clast fraction from Villalbeto de la Peña and Villalbeto de la Peña L-chondrite host matrix. For the Villalbeto de la Peña clast, the clast fraction was visually inspected for adhered matrix material and any visible matrix was removed by hand. Bulk powders were generated for each sample by crushing a fusion crust-free piece using an agate mortar and pestle. For two of the samples, Lundsgard and NWA 725, Cr-isotopic composition measurements were made using separated chromite grains instead of bulk powders. The bulk powders were homogenized and an aliquot of the powder (30–50 mg) was placed into a PTFE Parr bomb capsule with a 2:1 concentrated HF-HNO_3_ acid solution. For Lundsgard and NWA 725 the chromite grains were placed directly into their respective PTFE capsules with the 2:1 HF-HNO_3_ acid solution. The capsules were sealed in stainless steel jackets and heated in an oven for 96 h at 190 °C to ensure complete dissolution of all refractory phases. After dissolution, the sample solutions were dried down and treated with concentrated HNO_3_ and 6 N HCl to eliminate fluorides generated during the dissolution processes.

Chromium was removed from the sample matrix utilizing a 3-column chemistry procedure described in ref. 20. High-precision isotope ratios were measured in the purified Cr fractions using a Thermo Triton Plus thermal ionization mass spectrometer at the University of California at Davis. The Cr fractions were loaded onto outgassed tungsten filaments after being mixed with an Al-boric acid-silica gel activator solution. Each filament was loaded with 3 μg of Cr and four filaments were loaded for each sample for a total load of 12 μg. Each four filament set was bracketed with filaments loaded with the NIST SRM 979 Cr standard. The signal intensity for each filament was set to 10 V (±15%) for ^52^Cr with 10^11^Ω resistors. Each filament analysis consisted of 1,200 ratios with 8 s integrations times. A gain calibration was made at the beginning of each filament, and the baseline was measured and the amplifiers were rotated between each block of 25 ratios. The mass fractionation was corrected using an exponential law and a ^50^Cr/^52^Cr ratio of 0.051859 (ref. 21). The ^53^Cr/^52^Cr and ^54^Cr/^52^Cr ratios are expressed in ɛ-notation (that is, parts per 10,000 deviation from the NIST SRM 979 Cr standard).

### Oxygen isotopic analysis

Individual chromite grains, 50–100 μm in diameter, separated from Öst 65, were mounted in resin in 0.25-inch-diameter stainless steel bullets. Somewhat larger grains of Stillwater chromite standard were mounted in similar bullets. All grains were ground and polished to give a flat cross section at least 50 μm across. The grains were imaged with secondary electrons and measurement spots were selected to avoid cracks, surface imperfections, inclusions and so on. The spots were marked using the scanning electron microscope so that the spots could be seen by scanning ion imaging to accurately position the beam for measurement.

Oxygen-isotope compositions were measured using the University of Hawai‘i Cameca ims-1280 ion microprobe. The detailed methodology is provided in ref. 22. Here, we provide details specific for these measurements. A 1 nA Cs^+^ primary ion beam, focused to ∼10 μm, with a total impact energy of 20 keV was used. Spots were presputtered for 60 s using a 5-μm raster, after which the raster was reduced to 3 μm for measurement. Masses ^16^O^–^, ^17^O^–^ and ^18^O^–^ were measured simultaneously in multicollection mode. ^16^O^–^ and ^18^O^–^ were measured by multicollector Faraday cups with low-mass resolving power (MRP ∼2,000), while ^17^O^–^ was measured using the axial monocollector electron multiplier with MRP ∼5,600, sufficient to separate the ^16^OH^–^ interference. The relatively high ^17^O^–^ count rate produces a loss in gain due to aging of the first dynode of the electron multipler. To minimize this effect, the ^17^O^–^ signal was measured for only 4 s in each cycle, after which the beam was deflected into a monocollector Faraday cup for 10 s (procedure originally proposed by Kita *et al*.^23^). Signals for ^16^O^–^ and ^17^O^–^ collected during the 4-s interval were used to determine the ^17^O/^16^O ratio and the ^16^O^–^ and ^18^O^–^ signals collected during the 10-s interval were used to determine the ^18^O/^16^O ratio. Each measurement consisted of 30 cycles.

Measured data were corrected for detector background and deadtime (electron multiplier). The background corrections for the Faraday cups were made using a smoothed fit to the backgrounds measured during presputtering. A tail correction was made for the ^16^OH^–^ interference. The contribution of the ^16^OH^–^ tail at mass ^17^O was estimated from measurement of the low-mass tail of the ^17^O peak to be ∼6 × 10^-6^ of the ^16^OH^–^ interference. The height of the ^16^OH^–^ interference was measured at the end of each measurement. Even with our protocol to minimize changes in the gain of the electron multiplier, the gain did change with time. We checked the pulse-height distribution at the beginning of each day and set the gain to our ‘standard' condition. We did not check the gain again during the day, as we have learned that the act of checking the multiplier gain introduces a transient instability in the multiplier gain that compromises the data. The gain of the monocollector electron multiplier during the day was monitored by measuring the Stillwater chromite standard for five-six measurements at the beginning of each day, after five-six measurements of the unknown, and at the end of the day. The Stillwater measurements permitted us to model and account for the gain drift in the multiplier during each measurement day.

The uncertainties for the individual measurement data reported in Table 2 include the counting-statistical uncertainties from the individual measurements, the standard deviation of the standard measurements (including uncertainties in the drift correction in the case of ^17^O), and the uncertainties in the background and tail corrections. The mean values for Öst 65 are the error-weighted means and the standard errors for the set of measurements. The uncertainties do not include a systematic uncertainty associated with the deadtime correction for ^17^O. The deadtime of 30 ns has an uncertainty of ±1 nanosecond, which results in an uncertainty on δ^17^O and Δ^17^O of 0.2‰. When combined with the statistical errors for individual δ^17^O and Δ^17^O measurements, this uncertainty makes little difference. But this error cannot be incorporated into the calculation of the weighted mean and standard error, because it is a systematic error that is not reduced by making more measurements. It must be applied after the mean values and standard errors are calculated. When combined quadratically with the standard errors on the mean values, the total uncertainty for δ^17^O becomes ±0.27‰ and for Δ^17^O becomes ±0.29‰.

### Data availability

The authors declare that data supporting the findings of this study are available within the article.

## Additional information

**How to cite this article:** Schmitz, B. *et al*. A new type of solar-system material recovered from Ordovician marine limestone. *Nat. Commun.* 7:11851 doi: 10.1038/ncomms11851 (2016).

## Figures and Tables

**Table 1:** Chromium isotopic results for recent and fossil meteorites.*

**Meteorite name & sample**	ɛ^**53**^**Cr**	ɛ^**54**^**Cr**
Lundsgard	+0.21±0.04	−0.38±0.10
NWA 725	+0.05±0.04	−0.54±0.08
Öst 65 sample 1	+0.24±0.03	−0.23±0.07
Öst 65 sample 2	+0.23±0.04	−0.26±0.09
Öst 32	+0.21±0.04	−0.32±0.10
Öst 29	+0.19±0.05	−0.34±0.11
Öst 18	+0.31±0.04	−0.24±0.10
Villabeto de la Peña clast	+0.06±0.03	−0.52±0.10
Villabeto de la Peña host	+0.26±0.04	−0.29±0.11

^*^Errors for ɛ^53^Cr and ɛ^54^Cr values are 2σ SD.

**Table 2:** Oxygen isotopic results for Öst 65 chrome-spinel grains.*

**Grain and spot**	^**17**^**O/**^**16**^**O**	^**18**^**O/**^**16**^**O**
Grain 4, spot 1	0.00038301±0.00000017	0.0020113±0.0000009
Grain 4, spot 2	0.00038305±0.00000020	0.0020110±0.0000009
Grain 5, spot 1	0.00038329±0.00000016	0.0020117±0.0000009
Grain 8, spot 1	0.00038310±0.00000019	0.0020119±0.0000011
Grain 8, spot 2	0.00038317±0.00000021	0.0020118±0.0000010
Grain 9, spot 1	0.00038312±0.00000020	0.0020127±0.0000010
Grain 10, spot 1	0.00038306±0.00000017	0.0020119±0.0000012
		
Weighted mean and 2σ SD	0.00038311±0.00000007	0.0020117±0.0000004
Reduced χ^2^	1.226	1.178	

**Grain and spot**	**δ**^**17**^**O**	**δ**^**18**^**O**	Δ^**17**^**O**
Grain 4, spot 1	0.33±0.43	3.02±0.46	−1.24±0.50
Grain 4, spot 2	0.44±0.51	2.92±0.46	−1.07±0.57
Grain 5, spot 1	1.06±0.42	3.24±0.44	−0.62±0.47
Grain 8, spot 1	0.57±0.49	3.36±0.46	−1.18±0.55
Grain 8, spot 2	0.75±0.56	3.28±0.51	−0.96±0.62
Grain 9, spot 1	0.62±0.52	3.74±0.52	−1.32±0.59
Grain 10, spot 1	0.47±0.45	3.37±0.60	−1.28±0.55
			
Weighted mean and 2σ SD	0.61±0.18†	3.25±0.18	−1.08±0.21†
Reduced χ^2^	1.266	1.178	0.933

^*^Data are from measurement spots that are free of cracks, inclusions, overlaps onto other phases and so on that could have caused shifts in the oxygen-isotope ratios.

^†^Errors do not include the systematic uncertainty from the deadtime correction (∼±20‰). Including that uncertainty increases the errors on the mean values for δ^17^O and Δ^17^O to ±0.27 and ±0.29, respectively. The larger errors are used in Fig. 3. The errors on individual measurements are only minimally affected by the uncertainty in the deadtime.
